# Economic development and road traffic fatalities in Russia: analysis of federal regions 2004–2011

**DOI:** 10.1186/s40621-015-0051-6

**Published:** 2015-08-27

**Authors:** Huan He, Nino Paichadze, Adnan A. Hyder, David Bishai

**Affiliations:** 1 Department of Population, Family and Reproductive Health, Director, Interdepartmental Health Economics Program and Johns Hopkins International Injury Research Unit, Johns Hopkins Bloomberg School of Public Health, 615 N. Wolfe Street, Suite E8132, Baltimore, MD 21205 USA; 2 Department of International Health and Johns Hopkins International Injury Research Unit, Johns Hopkins Bloomberg School of Public Health, 615 N. Wolfe Street, Baltimore, MD 21205 USA

**Keywords:** Economic development, Road traffic fatalities, Crash fatality ratio, Road traffic injuries, Sub-national analysis, Russia

## Abstract

**Background:**

The relationship between economic development and road safety at sub-national level has not been well established. This study aims to assess the relationships between economic growth (measured by gross regional product (GRP)) and road traffic fatalities (RTFs) and crash fatality ratio (CFR) at sub-national level in Russia.

**Methods:**

We used published secondary data on annual RTFs and CFR obtained from the traffic police and socioeconomic development indicators from the statistics department for each Russian federal region (referred to in Russia as “subject”) for 2004–2011. We used multivariate fixed effects models for longitudinal data to examine the GRP-RTF and the GRP-CFR relationships excluding regions with extreme values. Time (in years) and a set of relevant socioeconomic variables (territory, population, number of privately owned cars, number of public buses, length of public motor roads, number of physicians, and budget expenditure on health care and physical wellness) were also included as covariates in the models.

**Results:**

The RTF rates decreased monotonically over time as GRP per capita increased in 66 studied regions during 2004–2011. This relationship was mainly explained by the number of privately owned cars and partially explained by year dummy variables, number of buses, and number of physicians. CFR also decreased monotonically as GRP per capita increased in 67 studied regions. This relationship between economic growth and CFR was fully explained by secular time trends. The year dummy effects on CFR were not mediated by other socioeconomic variables included in the study.

**Conclusions:**

For the period of 2004–2011 in Russia, the reduction in RTFs is mostly explained by increasing the number of private cars, while the reduction of CFR is mostly associated with year-effects suggesting a process of diffusion of knowledge, which is not solely dominated by economic growth.

## Background

Road traffic injuries are a leading cause of health burden globally (Chandran et al. 2010) and are projected to become the fifth leading cause of death by 2030 (World Health Organization 2008). In 2010, 80 % of the total road traffic fatalities (RTFs) occurred in middle-income countries, though population and registered vehicles in these countries respectively accounted for 72 and 52 % out of the world’s total (World Health Organization 2013). Middle-income countries had the highest annual RTF rates (20.1 per 100,000), and more than half of them were showing increases in the number of RTFs (World Health Organization 2013). Thus, reducing RTFs in middle-income countries is vital for reducing the global burden of road-traffic-related injuries and fatalities.

Previous studies on road safety have investigated the relationship between economic growth and RTFs. A number of these studies identified a non-linear (Van Beeck et al. 2000) or inverted U-shaped *Kuznets Curve* relationship (Kuznets 1955) between economic growth and RTFs. This has been shown in single country analyses based on data from India during 1999–2001 (Garg and Hyder 2006) and from Malaysia during 1979 to 2007 (Ahmat et al. 2011; Garg and Hyder 2006) and in multi-country analyses based on data from 1963 to 1999 (Kopits and Cropper 2005) and 1992 to 1996 (Bishai et al. 2006). The *Kuznets Curve* relationship suggests that RTFs increase with economic growth in lower-income settings and decrease in higher-income settings after the economic growth reaches a certain threshold. This indicates that economic growth may determine road safety in a region. However, some recent time-series studies have not shown the inverted U-shaped *Kuznets Curve*. Longitudinal studies in Oman (Al-Reesi et al. 2013) and Qatar (Bener et al. 2010), both high-income countries in the past three decades, actually found RTFs increased monotonically with economic growth. A cross-sectional exploration (Bhalla and Mohan, in preparation for publication) using recent RTFs data for all countries based on the 2010 Global Burden of Disease Study (Global Road Safety Facility et al. 2014) and the 2013 WHO Global Status Report on Road Safety (World Health Organization 2013) showed no clear relationship between gross national income (GNI) per capita and RTFs but revealed large variations of RTF status for countries of similar GNI per capita. These findings challenge the presence of a universally deterministic role of economic growth on road safety outcomes.

Bishai and colleagues have summarized four possible explanations for the *Kuznets Curve* of RTFs first rising then falling as economies grow, despite of the fact that economic grow usually accompanies with increasing exposure to road traffic transportation. These four explanations can be summarized as roads and regulation, competing risks, vehicle mix, and medical technology. The framework is as follows: (1) the *roads and regulation* explanation argues that more advanced phases of economic development are preconditions for the institutional capacity to regulate and intervene in road safety (Bishai et al. 2006). (2) The *competing risks* explanation assumes that lower-income countries (Bishai et al. 2003) underinvest in road safety and allocate generated income to other health risks (infectious and nutritional health risks), while higher-income countries, where such health risks recede, spend more money on road safety. The passage of time allows a reprioritization that brings road safety higher on a country’s agenda (Bishai et al. 2006). (3) The *vehicle mix* explanation assumes that economic growth spontaneously enables road users to shift to safer modes of transportation rather than to dangerous modes (e.g., motorcycles) in higher-income countries but not in lower-income countries (Bishai et al. 2006). (4) The *medical technology* hypothesis suggests that economic growth supports the development of more advanced pre-hospital and hospital trauma care systems in higher-income countries but not in lower-income countries (Bishai et al. 2006). A multivariate model analyses based on data from industrialized countries from the periods of 1972–2004 and 1970–1999 support the *roads and regulation* and *medical technology* explanations by showing that changes in institutional quality, implementation of road safety regulations, and medical care explain the *Kuznets Curve* for RTFs (Law et al. 2009, 2011).

Other perspectives for RTF prevention do not emphasize the deterministic role of economic development that much. For example, the “Haddon matrix” emphasizes more proximal determinants of road injuries without assuming that national income is required to alter these determinants. This perspective presumes that a country can just re-prioritize public spending for safety and take advantage of best practices without waiting for the national income to reach a certain threshold. A simulation study (Bhalla et al. 2007) using historical data of industrialized countries from 1963 to 1999 partially supports the “vehicle mix” explanation but shows that the *Kuznets Curve* for RTFs only occurs when motorization popularity is dominated by privately owned car use rather than “scooter use, bus use, and mixed use.”

A few studies have used cross-region longitudinal data within a single country to investigate economic growth and other determinates of RTFs to control for potential biases introduced by measurement heterogeneity and other unobserved variables that often appear in cross-country studies. Only two of these studies have been done in middle-income countries, one of which was in China when it moved from lower-middle-income country to upper-middle-income country during 1998 to 2006 (Wen 2008), while the other was in India when it remained as a lower-middle-income country during 1994–2006 (Grimm and Treibich 2013). However, these two studies were not conducted in a country during the transition from upper-middle to high-income stages, and they lacked major explanatory variables (e.g., indicators for health and trauma care capacity). Thus, the evidence based on sub-national analyses of middle-income countries is still scarce.

We focused on Russian Federation (Russia), a country that transitioned from upper-middle-income stage to high-income stage in the past decade (World Bank 2013). Russia is one of the ten leading countries contributing to road traffic fatalities (RTFs) in the world (World Health Organization 2009) and experiencing high economic costs from road traffic crashes (Marquez and Bliss 2010). In 2009, Mr. Dmitry Medvedev, a former President of Russia said: “The national economy lost $175 billion from road traffic accidents [crashes] over the past 5 years. That is comparable with overall health care expenditures of the same period” (Roth 2009). Russia has undergone rapid economic development and social change in recent years. In Russia, the gross national income (GNI) per capita (converted to international dollars using purchasing power parity rates) increased from US$10,030 in 2004 to US$21,860 in 2011 (World Bank 2014). The ownership of private cars almost doubled from 130.5 per 1000 persons in 2000 to 257.5 per 1000 persons in 2012 (Federal State Statistics Service of the Russian Federation 2012). The Russian government has raised the priority of road safety domestically and also endorsed international actions for road safety (Medvedev 2011). While more regulatory efforts have been directed towards reducing road traffic injuries and fatalities and controlling risk factors, such as the Federal Target Road Safety Program (2006–2012) (Breen et al. 2011), few studies have evaluated the effect of these social programs (Kudryavtsev et al. 2012; Pridemore et al. 2013). It is also important to note that there are no studies, which describe RTF trends at sub-national level or the relationship between economic development and RTFs in Russia.

Our study aims to explore the relationship between economic growth and RTFs in Russia using sub-national data from 2004 to 2011 and to investigate the effects of other major socioeconomic developments variables on RTFs. Our goal is to assess if other factors can explain the relationship between economic growth and RTFs. This study adds information to the discussion on the relationship between economic development and RTFs and identifies important socioeconomic variables that relate to changes in RTFs in Russia. We hope that our findings will facilitate tailoring ongoing and future road safety interventions in Russia and globally.

## Methods

### Study sample

The unit of analysis is a set of Russian federal regions, also referred to as “subject” in Russian governmental documents. Russian subjects are regional bodies of state authority of the Russian Federation and includes the following types of entities: Republic, Oblast (region), Krai (territory), Autonomous Oblast (autonomous region), Autonomous Okrug (autonomous area), and Federal City (Russian Federation 1993). There were 89 federal regions that existed through the period of 2004–2011 (Federal State Statistics Service of the Russian Federation 2013). However, 16 federal regions changed their administrative borderlines or subordination to provinces during the study period.^1^ To have comparable data across all federal regions throughout the study period, our study sample was limited to 73 federal regions where no boundary or other administrative changes occurred in 2004–2011, which led to 584 observed data points (8 annual repeated measures for each of the 73 regions). These 73 federal regions accounted for 93 % of the Russian population (Federal State Statistics Service of the Russian Federation 2013).

Seven federal regions with extreme values of major predictors (gross regional product (GRP) and number of private cars) and road safety outcomes were excluded from the analysis.^2^ We included 67 of 73 (dropped 6) Russian federal regions in our analyses of the GRP-RTF relationship and 66 of 73 (dropped 7) regions of the GRP-case fatality ratio (CFR) relationship through 2004–2011. The regions were mostly dropped due to extremely high GRP per capita (Moscow City, Neneck Autonomous Okrug, Tumensk Oblast, Yamalo-Nenecky Autonomous Okrug, Khanti-Mansiysky Autonomous Okrug, Chukotsk Autonomous Okrug), while only Tiva Republic was dropped due to extremely high CFR. After the adjustment, the analytic regions had lower average GRP per capita compared to the 73 eligible regions (Table 1) and lower than the national average GRP per capita (mean = 138,000 Russian rubles (RUB), median = 140,000 RUB, converted to the 2004 price value) during 2004–2011.

**Table 1:** Descriptive data of Russian federal regions, 2004–2011^a^

Variables	All eligible 73 (*n* = 584)	Studied 67 regions for GRP-RTF (*n* = 505)^b^	Studied 66 regions for GRP-CFR (*n* = 512)^c^
	Mean±SD	Mean±SD	Mean±SD
Road safety variables			
Road traffic fatality (RTF) rate, per 1000 person-years	0.2±0.08	0.2±0.06	0.2±0.07
Crash fatality ratio (CFR), fatalities per crash	0.2±0.05	0.1±0.04	0.1±0.03
Road traffic crash rate, per 1000 person-years	1.6±0.4	1.6±0.4	1.6±0.4
Road traffic injury rate, per 1000 person-years	2.0±0.6	2.0±0.5	2.0±0.5
Main socioeconomic variables			
Gross regional product (GRP) per capita, 1000 RUB	154.5±224.6	96.3±38.5	97.1±38.0
Territory, 1000 km^2^	237.5±487.4	194.1±442.9	191.7±440.2
Population, end of year, 1000 persons	1798.2±1688.9	1727.6±1262.5	1768.8±1324.2
Number of privately owned cars per 1000 persons	189.4±48.9	188.9±44.6	189.9±43.8
Number of public buses per 100,000 persons	49.2±30.8	44.3±22.1	44.8±22.2
Length of public motor roads with hard surface, 1000 km^d^	8.0±5.4	8.4±5.2	8.6±5.2
Number of physicians of all specialties, 1000	9.0±11.1	8.3±6.7	8.4±6.8
Consolidated budget expenditure on health care and physical wellness per capita, 1000 RUB	3.7±3.0	2.9±1.4	3.0±1.4

*RUB* Russian Ruble, *km* ^*2*^ square kilometer, *km* kilometer

^a^All monetary variables are converted to comparable price of 2004

^b^Observations were excluded for extreme values of GRP per capita (>226,000 RUB) or RTF rate (<53 or >326 per 1000 person-years)

^c^Observations were excluded for extreme values of GRP per capita (>226,000 RUB) or CFR (<0.04 or >0.25 fatalities per crash)

^d^No data of length of roads for Moscow City and St. Petersburg City

### Data source

For each Russian federal region, we obtained road safety data (numbers of road traffic crashes, injuries, and fatalities) from the traffic police (Federal State Statistics Service of the Russian Federation 2012) for the period of 2004–2011 and main socioeconomic data (GRP, population, territory, number of physicians, and consolidated budget expenditure on health care and physical wellness) from published sources (Federal State Statistics Service of the Russian Federation 2013) for 2004–2011 as well. We also used published data on transportation development (number of private cars, number of buses, length of public motor roads with hard surface) obtained from the Federal District Statistical Service (Federal State Statistics Service of the Russian Federation 2012).

### Variables

Police-reported annual counts of RTFs and CFR were used as major outcome variables. CFR was defined by number of fatalities over number of crashes. Annual counts of road traffic crashes and injuries were used as secondary outcomes for road safety. For descriptive analysis, we converted RTFs and road traffic crashes and injuries into annual rates per 1000 persons.

Economic growth of each Russian federal region was measured by GRP or the total value of all goods and services produced in a region (Federal State Statistics Service of the Russian Federation 2013). GRP for a region is similar to the concept of gross domestic product (GDP) for a country. The annual GRP for each Russian federal region during 2004–2011 was adjusted for inflation to comparable value in 2004.

Inclusion of other socioeconomic development variables was guided by the four reasons for the RTF Kuznets Curve discussed above (roads and regulation, competing risks, vehicle mix, and medical technology). First, density of hard surfaced motor roads was used as a proxy for *roads and regulation*. Second, time dummies were included to indicate how the priority of road safety fared against *competing risks* for policy makers. Time trends also indicate knowledge diffusion that could improve Russia’s ability to regulate transport safety. For instance, the Russian government requested a peer review of its performance on road safety from the Organization for Economic Co-operation and Development (OECD) committee in 2004 (European Conference of Ministers of Transport 2006). In 2006, Russia launched the national road safety program (Breen et al. 2011). There were also recent improvements in alcohol policy (Pridemore et al. 2013). Third, the number of privately owned cars and number of public buses were indictors of *vehicle mix.* Fourth, the number of physicians of all specialties and consolidated budget expenditure on health care were proxies for *medical technology* that could mitigate the odds of death after a road injury. Population and territory were also included to adjust for their differences cross regions. We chose these variables with reference to relevant studies (Bishai et al. 2006; Kopits and Cropper 2005; Law et al. 2011) and availability of data. Table 1 summarizes the distribution of all the included variables.

### Statistical analysis

First, we produced scatter plots of fatality rate, case fatality rate, crash rate, and injury rate against GRP per capita and time with locally weighted scatterplot smoothing (LOWESS) fitted lines of the overall relationship (Cleveland 1979; Lindsey and Sheather 2010). Then, we performed a series of multivariate longitudinal analyses of GRP on RTFs and CFR, adjusting for population and other socioeconomic variables (Bollen and Ward 1979; Schuessl 1974).

We used the following model:

1 $$ {Y}_{it}=C+{\beta}_1{X}_{it}+{\beta}_2Po{p}_{it}+{\mu}_i+{\varepsilon}_{it} $$

where *Y*_*it*_ is the count of the road safety-related health outcome; *X*_*it*_ is a set of covariates at *i*th region at time *t. Pop*_*it*_ is the population of that region at time *t*, and *μ*_*i*_ is a region-specific contribution to the error term. Both road safety and socioeconomic variables were entered in the model in their aggregate values. We controlled for population size by including population as a covariate in the regression model to avoid the well-known ratio bias that comes from dividing both dependent and independent variables by population size (Bollen and Ward 1979; Schuessl 1974). Both fixed effects and random effects models were fitted to compare their goodness-of-fit to the panel data for federal regions. Fixed effects models were ultimately chosen as the preferred ones, as the results from the Hausman tests (Hausman 1978) favored them over the random effects models for these data. Robust standard errors were produced for data clustering by federal region.

Exploratory analyses helped to select the final models. Data inspection revealed the presence of some extreme values for GRP. To control for this, we explored models that excluded regions with extreme values, particularly high GRP. Initial analyses used logarithm-transformed GRP and counts of road safety outcomes to control for the influence of long-tailed distribution, but this showed little improvement of model fitting. We also explored the use of quadratic terms of GRP to explore possible non-linear relationships between GRP and counts of the road safety-related health outcomes. However, the quadratic terms were not statistically significant and were thus excluded from the final model. Finally, the effect of including or excluding the variable of length of roads with hard surface was examined and the robustness of findings to various specifications was checked. This variable was excluded from final models as it wasn’t associated with fatality-related measures in any studied models, and its inclusion/exclusion did not change the other coefficients. All the data analyses were formed by STATA 12.0 software (College Station, TX, USA).

## Results

### Fatality characteristics

**Fig. 1:**
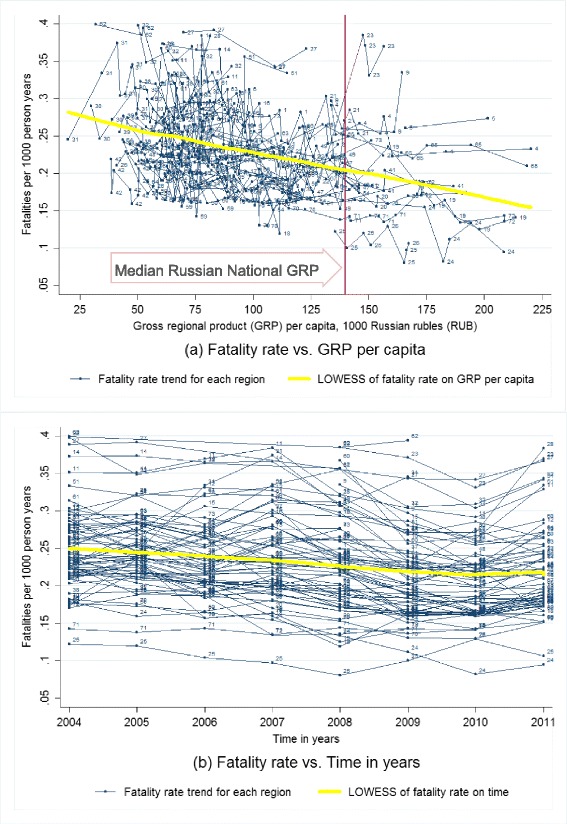
Road traffic fatality rate by gross regional product per capita and time for Russian federal regions, 2004–2011. **a** The *multiple thin lines* are connecting fatality rate for the same region from high to low gross regional product (GRP) per capita, to represent the regional original trends of fatality rate by GRP per capita; the *single thick yellow line* is the locally weighted scatterplot smoothing (LOWESS) non-parametric regression fitted curve of fatality rate on GRP per capita based on small intervals (width = 0.8) of GRP per capita, to represent the pooled original trend of fatality rate by GRP per capita; the *vertical reference line* is the median GRP per capita for whole Russia during 2004–2011. **b** The *multiple thin lines* are connecting fatality rate for the same region from early to late years, to represent the regional original trends of fatality rate by time; the *single thick yellow line* represents the locally weighted scatterplot smoothing (LOWESS) regression fitted curve of fatality rate on time in years based on small intervals (width = 0.8) of time in years, to represent the pooled original trends of fatality rate by time. 505 data points for 67 Russian federal regions at different years during 2004–2011 with non-outliers are included. Non-outliers are defined as GRP per capita <226 1000 RUB, fatality rate at 0.05–0.40 per 1000 person-years, and number of private cars at 53–326 per 1000 persons. Labels for data points: *1* Adygey Republic, *2* Altay Republic, *3* Altaysky Krai, *4* Amursk Oblast, *5* Arhangelsk Oblast, *6* Astrahan Oblast, *9* Bashkortastan Republic, *10* Belgorod Oblast, *11* Bryansk Oblast, *12* Buryatia Republic, *13* Chelyabinsk Oblast, *14* Chuvashskaya Republic, *15* Evresk Autonomous Oblast, *16* Irkutskaya Oblast, *17* Ivanovo Oblast , *18* Kaliningrad Oblast, *19* Kalmykia Republic, *20* Kaluzhsk Oblast, *21* Karelia Republic, *22* Kemerovskaya Oblast, *23* Khabarovsk Krai, *24* Khakasia Republic, *25* Kirovsk Oblast, *26* Komi Republic, *27* Kostroma Oblast, *28* Krasnodar Kray, *30* Krasnoyarsky Krai, *31* Kurgansk Oblast, *32* Kursk Oblast, *33* Leningrad Oblast, *34* Lipetsk Obalst , *35* Magadansk Oblast, *36* Mary El Republic, *37* Mordovia Republic, *38* Moscow Oblast, *39* Murmansk Oblast, *40* Nizhegorodsk Oblast, *41* Novgorod Oblast, *42* Novosibirskaya Oblast, *43* Omskaya Oblast, *44* Orenburg Oblast, *45* Orlov Oblast, *46* Pensa Oblast, *47* Primorsk Krai, *48* Pskov Oblast, *49* Rostovskaya Oblast, *50* Ryazan Oblast, *51* Saha Republic (Yakutia), *52* Sakhalin Oblast, *53* Samarsk Oblast, *54* Saratovsk Oblast, *56* Smolensk Oblast, *59* St. Petersburg City, *60* Sverdlovsk Oblast, *61* Tambovsk Oblast, *62* Tatarstan Republic, *63* Tiva Republic, *64* Tomskaya Oblast, *65* Tulsk Oblast, *66* Tver Oblast, *67* Udmurtskaya Republic, *68* Vladimir Oblast, *69* Volgogradkaya Oblast, *70* Vologda Oblast, *71* Voronezh Oblast, *72* Yaroslavl Oblast, *73* Ylianov Oblast

Descriptive scatterplots (Fig. 1a) show large variations of RTF rate and GRP per capita among different Russian regions. For most of the regions, RTF rate reduced as GRP per capita increased. The best-fit LOWESS curve indicated that RTFs decreased almost linearly as GRP per capita increased at the sub-national level across the analyzed 67 federal regions during 2004–2011. However, the LOWESS curve is not adjusted for any other covariates. Figure 1a shows that the majority of the studied regions were under the national median GRP per capita (the red vertical reference line with a label) for most of the years during 2004–2011. The pooled RTF rate (Fig. 1b) decreased over time, on average from about 0.25 per 1000 persons in 2004 to about 0.21 per 1000 persons in 2010, and remained flat until 2011.

### Crash fatality ratio characteristics

**Fig. 2:**
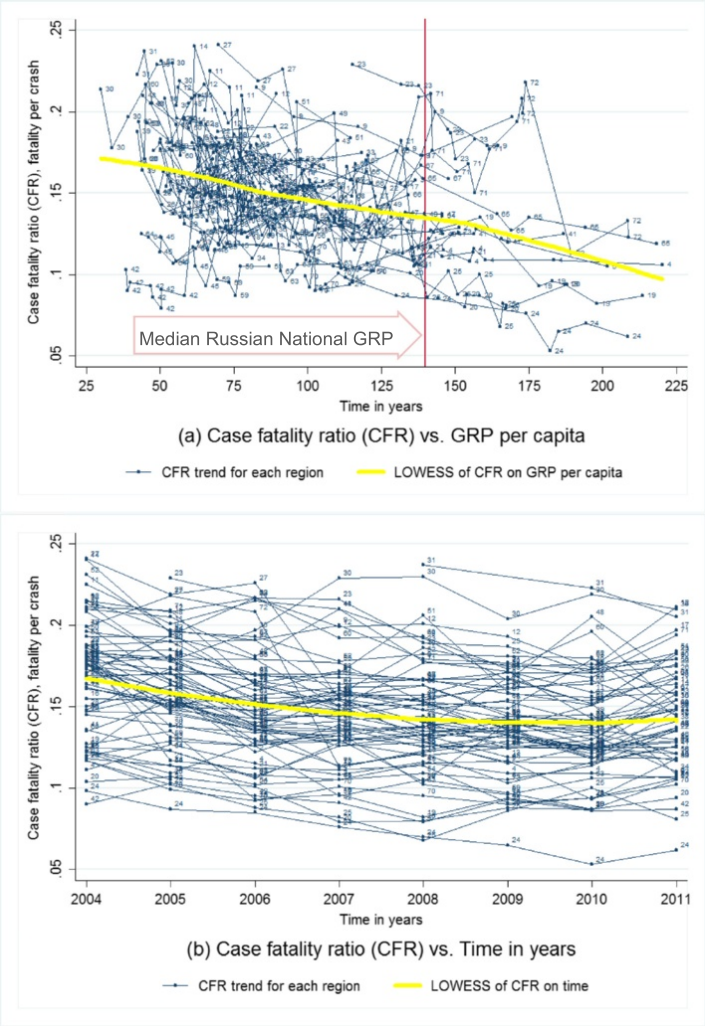
Case fatality ratio by gross regional product per capita and time for Russian federal regions, 2004–2011. **a** The *multiple thin lines* are connecting case fatality ratio (CFR) for the same region from high to low gross regional product (GRP) per capita, to represent the regional original trends of CFR by GRP per capita; the *single thick yellow line* is the locally weighted scatterplot smoothing (LOWESS) non-parametric regression fitted curve of CFR on GRP per capita based on small intervals (width = 0.8) of GRP per capita, to represent the pooled original trend of CFR by GRP per capita; the *vertical reference line* is the median GRP per capita for whole Russia during 2004–2011. **b** The *multiple thin lines* are connecting CFR for the same region from early to late years, to represent the regional original trends of CFR by time; the *single thick yellow line* represents the locally weighted scatterplot smoothing (LOWESS) regression fitted curve of CFR on time in years based on small intervals (width = 0.8) of time in years, to represent the pooled original trends of CFR by time. 512 data points for 66 Russian federal regions at different years during 2004–2011 with non-outliers are included. Non-outliers are defined as GRP per capita <226 1000 RUB, CFR at 0.04–0.25, and number of private cars at 53–326 per 1000 persons. Labels for data points: *1* Adygey Republic, *2* Altay Republic, *3* Altaysky Krai, *4* Amursk Oblast, *5* Arhangelsk Oblast, *6* Astrahan Oblast, *9* Bashkortastan Republic, *10* Belgorod Oblast, *11* Bryansk Oblast, *12* Buryatia Republic, *13* Chelyabinsk Oblast, *14* Chuvashskaya Republic, *15* Evresk Autonomous Oblast, *16* Irkutskaya Oblast, *17* Ivanovo Oblast , *18* Kaliningrad Oblast, *19* Kalmykia Republic, *20* Kaluzhsk Oblast, *21* Karelia Republic, *22* Kemerovskaya Oblast, *23* Khabarovsk Krai, *24* Khakasia Republic, *25* Kirovsk Oblast, *26* Komi Republic, *27* Kostroma Oblast, *28* Krasnodar Kray, *30* Krasnoyarsky Krai, *31* Kurgansk Oblast, *32* Kursk Oblast, *33* Leningrad Oblast, *34* Lipetsk Obalst , *35* Magadansk Oblast, *36* Mary El Republic, *37* Mordovia Republic, *38* Moscow Oblast, *39* Murmansk Oblast, *40* Nizhegorodsk Oblast, *41* Novgorod Oblast, *42* Novosibirskaya Oblast, *43* Omskaya Oblast, *44* Orenburg Oblast, *45* Orlov Oblast, *46* Pensa Oblast, *47* Primorsk Krai, *48* Pskov Oblast, *49* Rostovskaya Oblast, *50* Ryazan Oblast, *51* Saha Republic (Yakutia), *52* Sakhalin Oblast, *53* Samarsk Oblast, *54* Saratovsk Oblast, *56* Smolensk Oblast, *59* St. Petersburg City, *60* Sverdlovsk Oblast, *61* Tambovsk Oblast, *63* Tatarstan Republic, *64* Tomskaya Oblast, *65* Tulsk Oblast, *66* Tver Oblast, *67* Udmurtskaya Republic, *68* Vladimir Oblast, *69* Volgogradkaya Oblast, *70* Vologda Oblast, *71* Voronezh Oblast, *72* Yaroslavl Oblast, *73* Ylianov Oblast

According to descriptive analyses based on scatterplots (Fig. 2a), large regional variations of CFR and declines in CFR are sustained as GRP per capita increases at the sub-national level across analyzed federal regions during 2004–2011. However, compared to results of RTF rate (Fig. 1a), there seemed to be even larger variations of CFR when GRP per capita was low (see the left half of both Fig. 2a, b) and more shape declines of CFR when GRP per capita was high (see the right half of both Fig. 2a, b). Similar to time tends of RTFs, CFR also decreased on average from about 170 fatalities out of 1000 crashes (CFR = 0.17) in 2004 to about 140 fatalities out of 1000 crashes (CFR = 0.14) in 2009 and stayed flat (Fig. 2b). However, the regional and pooled trends decline faster for CFR as compared to RTF rate from the period from 2004 to about 2007 or 2008.

### Crash and injury characteristics

**Fig. 3:**
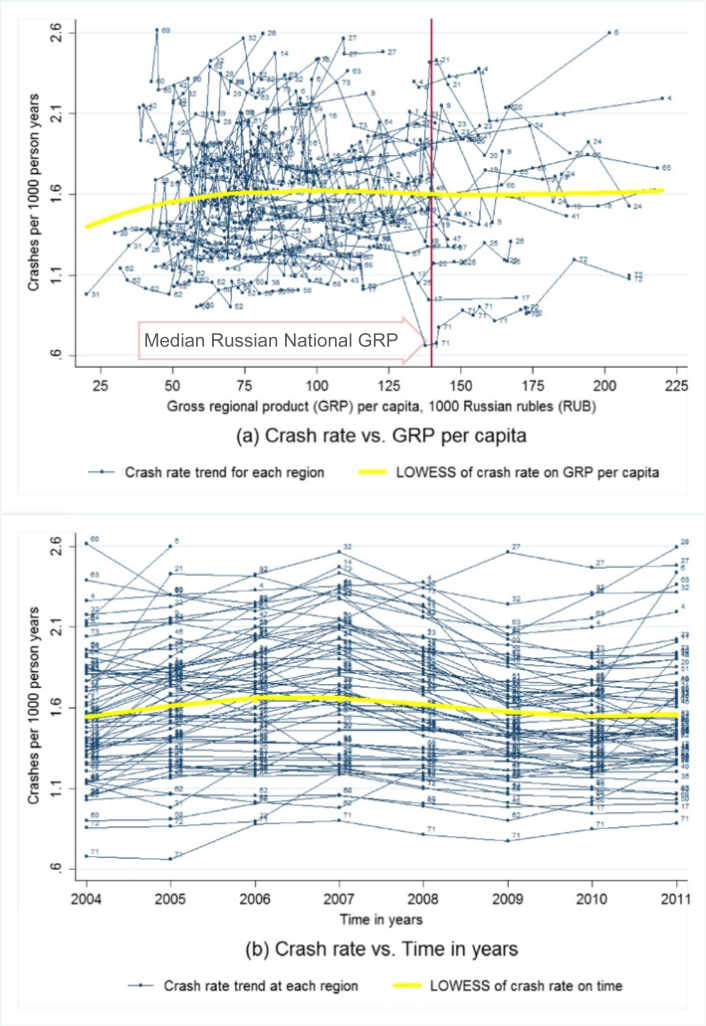
Road traffic crash rate by gross regional product per capita and time for Russian federal regions, 2004–2011. **a** The *multiple thin lines* are connecting crash rate for the same region from high to low gross regional product (GRP) per capita, to represent the regional original trends of crash rate by GRP per capita; the *single thick yellow line* is the locally weighted scatterplot smoothing (LOWESS) non-parametric regression fitted curve of crash rate on GRP per capita based on small intervals (width = 0.8) of GRP per capita, to represent the pooled original trend of crash rate by GRP per capita; the *vertical reference line* is the median GRP per capita for whole Russia during 2004-2011. **b** The *multiple thin lines* are connecting crash rate for the same region from early to late years, to represent the regional original trends of crash rate by time; the *single thick yellow line* represents the locally weighted scatterplot smoothing (LOWESS) regression fitted curve of crash rate on time in years based on small intervals (width = 0.8) of time in years, to represent the pooled original trends of crash rate by time. 524 data points for 67 Russian federal regions at different years during 2004–2011 with non-outliers are included. Non-outliers are defined as GRP per capita <226 1000 RUB, crash rate at 0.4–2.7 per 1000 person-years, and number of private cars at 53–326 per 1000 persons. Labels for data points: *1* Adygey Republic, *2* Altay Republic, *3* Altaysky Krai, *4* Amursk Oblast, *5* Arhangelsk Oblast, *6* Astrahan Oblast, *9* Bashkortastan Republic, *10* Belgorod Oblast, *11* Bryansk Oblast, *12* Buryatia Republic, *13* Chelyabinsk Oblast, *14* Chuvashskaya Republic, *15* Evresk Autonomous Oblast, *16* Irkutskaya Oblast, *17* Ivanovo Oblast , *18* Kaliningrad Oblast, *19* Kalmykia Republic, *20* Kaluzhsk Oblast, *21* Karelia Republic, *22* Kemerovskaya Oblast, *23* Khabarovsk Krai, *24* Khakasia Republic, *25* Kirovsk Oblast, *26* Komi Republic, *27* Kostroma Oblast, *28* Krasnodar Kray, *30* Krasnoyarsky Krai, *31* Kurgansk Oblast, *32* Kursk Oblast, *33* Leningrad Oblast, *34* Lipetsk Obalst , *35* Magadansk Oblast, *36* Mary El Republic, *37* Mordovia Republic, *38* Moscow Oblast, *39* Murmansk Oblast, *40* Nizhegorodsk Oblast, *41* Novgorod Oblast, *42* Novosibirskaya Oblast, *43* Omskaya Oblast, *44* Orenburg Oblast, *45* Orlov Oblast, *46* Pensa Oblast, *47* Primorsk Krai, *48* Pskov Oblast, *49* Rostovskaya Oblast, *50* Ryazan Oblast, *51* Saha Republic (Yakutia), *52* Sakhalin Oblast, *53* Samarsk Oblast, *54* Saratovsk Oblast, *56* Smolensk Oblast, *59* St. Petersburg City, *60* Sverdlovsk Oblast, *61* Tambovsk Oblast, *62* Tatarstan Republic, *63* Tiva Republic, *64* Tomskaya Oblast, *65* Tulsk Oblast, *66* Tver Oblast, *67* Udmurtskaya Republic, *68* Vladimir Oblast, *69* Volgogradkaya Oblast, *70* Vologda Oblast, *71* Voronezh Oblast, *72* Yaroslavl Oblast, *73* Ylianov Oblast

**Fig. 4:**
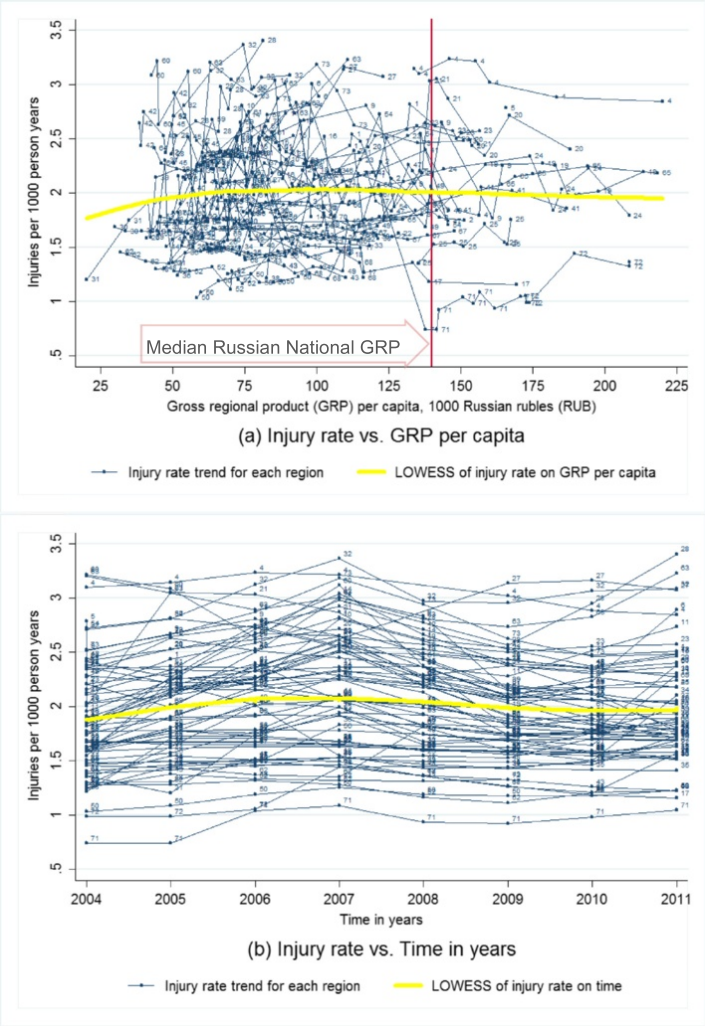
Road traffic injury rate by gross regional product per capita and time for Russian federal regions, 2004-2011. **a** The *multiple thin lines* are connecting injury rate for the same region from high to low gross regional product (GRP) per capita, to represent the regional original trends of injury rate by GRP per capita; the *single thick yellow line* is the locally weighted scatterplot smoothing (LOWESS) non-parametric regression fitted curve of injury rate on GRP per capita based on small intervals (width = 0.8) of GRP per capita, to represent the pooled original trend of injury rate by GRP per capita; the vertical reference line is the median GRP per capita for whole Russia during 2004–2011. **b** The *multiple thin lines* are connecting injury rate for the same region from early to late years, to represent the regional original trends of injury rate by time; the *single thick yellow line* represents the locally weighted scatterplot smoothing (LOWESS) regression fitted curve of injury rate on time in years based on small intervals (width = 0.8) of time in years, to represent the pooled original trends of injury rate by time. 522 data points for 67 Russian federal regions at different years during 2004–2011 with non-outliers are included. Non-outliers are defined as GRP per capita <226 1000 RUB, injury rate at 0.3–3.5 per 1000 person-years, and number of private cars at 53–326 per 1000 persons. Labels for data points: *1* Adygey Republic, *2* Altay Republic, *3* Altaysky Krai, *4* Amursk Oblast, *5* Arhangelsk Oblast, *6* Astrahan Oblast, *9* Bashkortastan Republic, *10* Belgorod Oblast, *11* Bryansk Oblast, *12* Buryatia Republic, *13* Chelyabinsk Oblast, *14* Chuvashskaya Republic, *15* Evresk Autonomous Oblast, *16* Irkutskaya Oblast, *17* Ivanovo Oblast , *18* Kaliningrad Oblast, *19* Kalmykia Republic, *20* Kaluzhsk Oblast, *21* Karelia Republic, *22* Kemerovskaya Oblast, *23* Khabarovsk Krai, *24* Khakasia Republic, *25* Kirovsk Oblast, *26* Komi Republic, *27* Kostroma Oblast, *28* Krasnodar Kray, *30* Krasnoyarsky Krai, *31* Kurgansk Oblast, *32* Kursk Oblast, *33* Leningrad Oblast, *34* Lipetsk Obalst , *35* Magadansk Oblast, *36* Mary El Republic, *37* Mordovia Republic, *38* Moscow Oblast, *39* Murmansk Oblast, *40* Nizhegorodsk Oblast, *41* Novgorod Oblast, *42* Novosibirskaya Oblast, *43* Omskaya Oblast, *44* Orenburg Oblast, *45* Orlov Oblast, *46* Pensa Oblast, *47* Primorsk Krai, *48* Pskov Oblast, *49* Rostovskaya Oblast, *50* Ryazan Oblast, *51* Saha Republic (Yakutia), *52* Sakhalin Oblast, *53* Samarsk Oblast, *54* Saratovsk Oblast, *56* Smolensk Oblast, *59* St. Petersburg City, *60* Sverdlovsk Oblast, *61* Tambovsk Oblast, *62* Tatarstan Republic, *63* Tiva Republic, *64* Tomskaya Oblast, *65* Tulsk Oblast, *66* Tver Oblast, *67* Udmurtskaya Republic, *68* Vladimir Oblast, *69* Volgogradkaya Oblast, *70* Vologda Oblast, *71* Voronezh Oblast, *72* Yaroslavl Oblast, *73* Ylianov Oblast

According to descriptive analyses based on scatterplots (Figs. 3a and 4a), the number of road traffic crashes and injuries is stable with both time and economic growth for most of the studied Russian federal regions during 2004–2011. We did not observe any bivariate relationships between numbers of either crashes or injuries with GRP per capita even in binary analyses, except for some increases driven by one region with the lowest GRP per capita. The crash rate and injury rate were mostly the same during 2004–2011 (Figs. 3b and 4b), except for some increases during 2004–2006 and the following decreases during 2007–2009. Therefore, number of crashes and injuries were not used for fixed effects model analysis.

### Association between economic growth and fatalities/crash fatality ratio: fixed effects models

The effect of economic growth on RTFs and CFR was further examined in multivariate fixed effects models (Table 2). Model 1 (the basic model) showed that there was a 0.690-point reduction in the number of fatalities per unit increase in GRP across 67 federal regions during 2004–2011, adjusted for population and territory. Model 2 showed the effect of GRP on RTFs was attenuated when other socioeconomic development variables were added to the model, especially time dummies and number of physicians. In model 3, however, the effect of GRP lost its statistical significance when the number of privately owned cars was controlled for in the model. In addition, according to the final model (model 3), not all time periods had significant effects on RTFs. The most significant effects were in 2006, 2009, and 2010.

**Table 2:** Multivariate fixed effects models on road traffic fatalities and crash fatality ratio

	Road traffic fatalities (count)			Crash fatality ratio (fatalities per 1000 crashes)		
	Model 1	Model 2	Model 3	Model 4	Model 5	Model 6
	*ß* (SD)	*ß* (SD)	*ß* (SD)	*ß* (SD)	*ß* (SD)	*ß* (SD)
GRP, bln RUB	−0.690***	−0.405***	0.115	−0.139***	0.010	0.007
	[0.055]	[0.079]	[0.074]	[0.018]	[0.021]	[0.035]
Population, 1000	0.065	−0.082	0.022	0.098***	−0.019	−0.003
	[0.059]	[0.063]	[0.053]	[0.020]	[0.020]	[0.025]
Territory, 1000 km^2^	0.016	0.054	−0.070	0.364	0.295	0.336
	[0.731]	[0.582]	[0.477]	[0.526]	[0.451]	[0.455]
Year						
2004 (reference)		0.000	0.000		0.000	0.000
		[0.000]	[0.000]		[0.000]	[0.000]
2005		−2.902	1.762		−9.878***	−10.113***
		[6.768]	[5.559]		[2.642]	[2.697]
2006		−20.309***	−13.928**		−21.393***	−21.814***
		[7.237]	[5.951]		[2.776]	[2.875]
2007		−7.030	4.336		−25.763***	−26.145***
		[7.807]	[6.451]		[2.998]	[3.109]
2008		−30.853***	−7.552		−28.997***	−29.259***
		[8.068]	[6.810]		[3.152]	[3.309]
2009		−70.890***	−29.258***		−32.199***	−32.299***
		[7.540]	[6.824]		[2.932]	[3.354]
2010		−80.318***	−31.181***		−34.196***	−34.130***
		[7.949]	[7.355]		[3.071]	[3.586]
2011		−49.251***	4.712		−29.364***	−28.922***
		[8.668]	[8.033]		[3.361]	[3.894]
Public buses, 1000		−3.703	−24.666***			−3.692
		[8.887]	[7.431]			[3.408]
Physicians, 1000		−22.358***	−6.132			−0.109
		[7.181]	[5.995]			[2.810]
Health budget, bln RUB		2.458	1.232			0.187
		[1.632]	[1.341]			[0.614]
Private cars, 1000			−0.657***			−0.007
			[0.046]			[0.022]
Constant	386.369**	791.011***	630.954***	−70.153	144.558	114.844
*R* ^2^ within	0.27	0.558	0.703	0.138	0.378	0.380
Additive *R* ^2^ within	0.27	0.288	0.145	0.138	0.24	0.002
Observations number	505	505	505	512	512	512
Regions number	67	67	67	66	66	66
*Ρ*(Rho)	0.981	0.996	0.997	0.993	0.990	0.992
Adjusted *R* ^2^	0.971	0.982	0.988	0.750	0.816	0.815

Length of road not significant to be included in the final models

*ß* (*SD*) coefficient from linear fixed effects model and its standard deviation, *bln* billion, *RUB* Russian ruble, *km* ^*2*^ square kilometer, *km* kilometer, *R* ^*2*^ coefficient of determination

***p < 0.001; **p < 0.01; **p < 0.01 to **p < 0.05

For CFR, model 4 showed that CFR decreased linearly as GRP increased during 2004–2011. Model 5 showed that time dummies explained all of the effect of economic growth on reduction in CFR. Model 6 showed that neither the number of public buses nor privately owned cars, nor the number of physicians or the health budget had statistically significant effect on CFR. According to the final model (model 6), the effects of time on CFR were still significant when other socioeconomic variables were controlled, though the declines were not even over the years. The largest annual drop in CFR occurred from 2005 to 2006 (−11.7 fatalities per 1000 crashes), followed by the drop from 2004 to 2005 (−10.1 fatalities per 1000 crashes) (Table 2, model 6).

## Discussion

In our sub-national analysis for Russia, we found that the rate of RTFs decreased linearly with economic growth, but the rates of road traffic injuries and crashes were not statistically related to economic growth. Previous literature has found that the inverted U-shaped *Kuznets Curve* relationship with economic growth only applied to RTFs, while road traffic crashes and injuries were shown to rise monotonically with increasing national income (Bishai et al. 2006). Several considerations might explain the pattern found in Russia. First, Russian federal regions may have already passed the peak of the *Kuznets Curve* for RTFs before Russia became an upper-middle-income country (World Bank 2013). Second, the expected increasing trend of RTFs might have been interrupted by the 2008–2009 Russian financial crisis (Council on Foreign Relations 2009; Cheung et al. 2009), during which period transport volume might have declined (Stuckler et al. 2009). Also, there might be a difference in patterns occurring in multi-country studies compared to sub-national analysis; if financing of transport administration, health care, and road safety activities are heavily centralized, influence of regional economic performance on road safety indicators might be less noticeable (Bishai et al. 2003).

It is interesting to observe a declining trend of CFR among studied Russian federal regions that could not be explained by any of the macro-variables of interest, including regional economic performance or the number of privately owned vehicles. The national level CFR was about 13.3–16.5 deaths per 100 crashes in Russia during 2004–2011, which was higher than in the United Kingdom (1.4–1.8) and Sri Lanka (11.7–13.6) during 1997–2002 (Mishra et al. 2010) but lower than in China during 2004–2006 (21–24) (Wen 2008). We have been unable to find prior longitudinal studies on the effect of economic growth on CFR, but there is a cross-sectional study based on data from 83 countries which showed that the injury fatality ratio, which is conceptually close to CFR, decreased as the gross national product increased in 1990 (Soderlund and Zwi 1995). This result is consistent with the results from our analysis when the effect of time was not controlled. In addition, given the fact that the road traffic crash rate have been stable during the study period and RTFs are a product of road traffic crash rate and CFR, our study implies that CFR is the major contributor to the downward trend of RTF rate observed recently in Russia.

CFR and transport fatality counts reflect different aspects of the safety environment. CFR reflects qualitative changes in the lethality of crashes. CFR can indicate how much energy is transferred to humans in each crash, and additionally, it reflects the effectiveness of post-crash care. Fatality counts reflect both lethality of crashes and the numbers of crashes as influenced by vehicle, roadway, and driver factors prior to and during the crash event as suggested by the Haddon matrix (Haddon 1968; Peden et al. 2004). The conceptual differences of these two measures were also reflected in our results, which showed that CFR captured more variability among regions even in low-income settings and was a more sensitive indicator of the secular changes of knowledge diffusion and policy changes, while RTFs were more sensitive to exposure to crashes and motorization. This distinction can help identify effective safety promotion interventions for countries at different development stages.

Among other socioeconomic development covariates included in our study, increasing numbers of privately owned cars explained most of the decreasing trend of RTFs in Russia during 2004–2011. This result is discordant with the analyses that included lower- and middle-income countries which found that RTFs increased as logarithm of vehicle per capita increased in 60 countries during 1972–2004 (Law et al. 2011) and in 41 countries during 1992–1996 (Bishai et al. 2006). Our results are similar to a sub-national analysis done in the Netherlands during 1982–1984 (Van Beeck et al. 1991). The observed negative effect of the increasing number of privately owned cars could be caused by various transportation development processes. First, more vehicles may increase the density of traffic flow and road congestion can decrease the speed of vehicle movement and reduce severity of crashes. Second, greater density can sometimes be simultaneous with improvements in infrastructure and enforcement, as was seen in one study in Arkhangelsk area of Russia during 2006–2010 (Kudryavtsev et al. 2012). Third, more vehicles might signify an overall switch to safer transport modes, such as using cars with improved safety measures or moving away from two wheelers to private cars induced by the motorization process (Bhalla et al. 2007), or by a fewer non-motorized travelers (especially pedestrians) (Paulozzi et al. 2007), or by higher proportion of vehicles with better safety features. However, the effect of number of privately owned cars could also be confounded by other factors that were not addressed by available data, such as improved design and quality of cars or more careful drivers. Further studies are needed to understand and acknowledge the effects of the undergoing rapid motorization in Russia.

In our study, numbers of physicians partially explained the association between economic development and RTFs, but consolidated budget expenditures on health care and physical wellness were not associated with RTFs. These results are somewhat consistent with findings from previous studies (Law et al. 2011; Van Beeck et al. 1991), which suggests that general advancements in medical care reduce RTF rate. Unfortunately, we were not able to assess how much the improved trauma care capacity contributes to the improved RTF rate and CFR in Russia due to the unavailability of specific geographic data on the Russian trauma system.

The simple passage of years stands out as one of the most important variables that contribute to the changes in road fatality outcomes in Russia. This is consistent with the results from a sub-national analysis in India (Grimm and Treibich 2013). As mentioned earlier, year dummy effects could reflect secular changes in knowledge diffusion, policy changes, and economic crises. During our study period in Russia, the most dramatic change of CFR and RTFs rate occurred in 2004–2006. These changes are in line with the timing of a peer review of road performance process requested by Russian government in 2004 (European Conference of Ministers of Transport 2006) and the development and implementation of the Federal Target Road Safety Program 2006–2012 (Breen et al. 2011) and alcohol control policies effective as of 2006 (Pridemore et al. 2013). The 2004 peer review report not only increased awareness of Russia’s poor performance on road safety but also provided a set of localized recommendations for Russian government based on evidence and experience from other countries, which were used to develop the road safety policies launched since 2006. Results of some evaluation studies have confirmed the effectiveness of the road safety policies in Russia. A regional evaluation suggested that the improvement of infrastructure (e.g., more signalized crosswalks) and increase in the penalties for road traffic violations by pedestrians and drivers who ignore prohibiting signals were associated with significant reduction in car-pedestrian crashes (Kudryavtsev et al. 2012). Another national evaluation study found an 11 % reduction in RTFs in males after enforcing the alcohol policies of 2006 (Pridemore et al. 2013). In our sub-national analysis, we found that the number of passenger violations was negatively associated with the decreased RTF rate in the Russian federal regions during 2008–2011, but this weak association disappeared when controlled for other key socioeconomic variables. Thus, further qualitative and quantitative studies are needed to better understand the time trend of RTF rate and CFR in Russia.

Our study is the first which uses sub-national panel data to study the characteristics of RTFs and CFR in Russia. The strengths of our study lie in the range of outcomes and covariate, and the attention to time trends in RTF. Police recorded road safety data were usually underreporting the number of RTFs compared to hospital-treated road traffic injuries (Elvik and Mysen 1999) and more likely to capture fatalities of males, drivers, or pedestrians and fatalities from incidents involving with more than one vehicle (Samuel et al. 2012), but RTFs data from the police are likely to be more reliable than measuring RTFs by hospitals in Russia. A study evaluated RTF data from these two sources and health-care based reports of injuries were found to be higher than police-data reports (Kudryavtsev et al. 2013). Our study had some limitations. First, our observations spanned across a relatively short time period (8 years) and excluded the extremely wealthy regions of Russia, which limited us to observe long-term dynamics and apply our findings to wealthy regions. However, the exclusion of extremely wealthy regions made our analytic samples more relevant to middle-income countries. Second, we failed to include some important covariates in the model due to missing data for some variables, such as the number of traffic violations before 2008, and unavailability of other variables, such as capacity of emergency care, alcohol consumption, speeding, or seatbelt use data. This could lead to bias due to unobservable confounders, which would have limited our ability to explain the relationship between economic development and RTFs. Third, although statistical analyses were carefully performed using fixed effects models, these are insufficient to make conclusions on causal relationships.

## Conclusions

Our study found that RTFs decreased monotonically with economic growth in most Russian federal regions during 2004–2011, but more detailed analysis revealed that the decreases of RTFs were primarily explained by secular time trends, the number of privately owned cars, and secondarily explained by the number of buses and number of physicians. This study also found that CFR significantly decreased during this period through a process dominated by the secular trend, which was not explained economic growth or other socioeconomic variables. In sum, road safety performance at sub-national level was not solely dominated by economic growth levels but a result of development in multiple processes, including motorization, knowledge diffusion and policy changes, and health-care capacity.

For future studies, we recommend collecting more relevant data on potential determinants (e.g., knowledge diffusion and policy enforcement) to understand why Russia successfully decreased RTFs and CFR in a period with rapid motorization, which challenges most middle-income countries in the world. We urge researchers and policy makers to use a systematic and comprehensive framework to investigate and evaluate road safety outcomes and performance. It is important not to rely too heavily on a deterministic relationship between economic growth and RTFs. Our results lead us to reject the premise that waiting for economic growth to take care of road safety is the right approach. In this new era, easier accesses to knowledge and technology in road safety and trauma care systems may change the landscape of road safety practices in low- and middle-income countries. Our results also highlight the usefulness of the CFR as a helpful indicator of post-crash road safety.

## Footnotes

1 The following federal regions were eliminated from analysis because their boundaries changed or belonged to different Federal Okrug during the study period: Republic of Dagestan, Republic of Ingushetia, Republic of Kabardino-Balkaria, Republic of Karachay-Cherkess, Republic of North Ossetia, Chechen Republic, Stavropolsky Krai, Permsky krai, Permskaya Oblast, Komi-Permyak Autonomous Okrug, Zabaykalsky Krai, Chita oblast, Agin-Buryat Autonomous Okrug, Taymyr Autonomous Okrug, Ust-Ordynsky Buryatsky Autonomous Okrug, Evenk Autonomous Okrug, Kamchatsky Krai, Kamchatskaya Oblast, and Koryak Autonomous Okrug.

2 Extreme values were defined as values beyond median plus two interquartile ranges (IQR) or below median minus two IQRs for each variable based on distribution of 584 data points (73 regions * 8 years), extreme values of GRP per capita during the study period defined as >226,000 RUB, extreme RTF rates defined as <53 or >326 per 1000 persons, extreme CFR values defined as <0.04 or >0.25 fatalities per crash, extreme values of number of privately owned cars defined as <53 or >326 cars per thousand persons.
